# Hereditary Diffuse Gastric Cancer: More than What Meets the Endoscopic Eye

**Published:** 2018-11-29

**Authors:** Muhammad Aziz, Rashna Madan, Ajay Bansal

**Affiliations:** 1 Department of Internal Medicine, University of Kansas Medical Center, Kansas City, KS; 2 Department of Pathology, University of Kansas Medical Center, Kansas City, KS; 3 Department of Gastroenterology and Hepatology, University of Kansas Medical Center, Kansas City, KS

**Keywords:** gastric neoplasms, familial diffuse gastric cancer, signet ring cell carcinoma, adenocarcinoma, gastrectomy

A 39-year-old female underwent esophagogastroduodenoscopy (EGD) after she was found to have hereditary diffuse gastric cancer (HDGC). Seven of patient’s family members were diagnosed with gastric adenocarcinoma between 20 – 40 years of age. Genetic testing revealed CDH-1 mutation. Endoscopic findings revealed normal esophagus, gastroesophageal junction, and stomach (Figure 1). A total of 30 biopsies (six each from antrum, distal stomach, transition zone, proximal stomach, and fundus) were obtained per Cambridge protocol.^1^ One of 30 biopsies demonstrated a focus of intramucosal adenocarcinoma in the fundus (Figure 2). The patient underwent a total gastrectomy with esophagojejunostomy. The gross specimen that was morphologically normal showed a total of 19 foci (Figure 3; rectangles) of poorly differentiated intramucosal adenocarcinoma ranging from 0.5 – 2.5 mm (Figures 4 and 5); 11 foci in the fundus and 8 foci in the body. The final stage was 1A (T1a, N0, M0) diffuse signet-ring carcinoma.

## DISCUSSION

Hereditary diffuse gastric cancer is associated with mutation in CDH-1 gene that encodes for tumorsuppressor protein E-cadherin.^2^ The cumulative risk of gastric cancer with known mutation in CDH-1 is reported to be 70% (95% CI 59 – 80%) and 56% (95% CI 44 – 69%) for men and women respectively.^3^ The average age of onset for stomach cancer with this mutation is 38 years (range 14 – 69 years) compared to general population which is usually between 60 – 80 years.^4^,^5^ The incidence of lobulated breast cancer in women is 42 % harboring this mutation.^5^ Endoscopic surveillance can be low yield if the tumor is microscopic and is not sampled with random biopsies during EGD. In our patient, endoscopic biopsies missed 94% (18/19) of the foci of intramucosal cancer. This case highlighted limitations of gastric biopsies in surveillance of gastric cancer in HDGC. Clinicians should strongly consider total gastrectomy in patients with HDGC because endoscopic surveillance has a high miss rate. If endoscopic surveillance is pursued, the Cambridge protocol with at least 30 biopsies should be followed and patient should be educated about the hazards of delaying surgical resection.

## Figures and Tables

**Figure 1:**
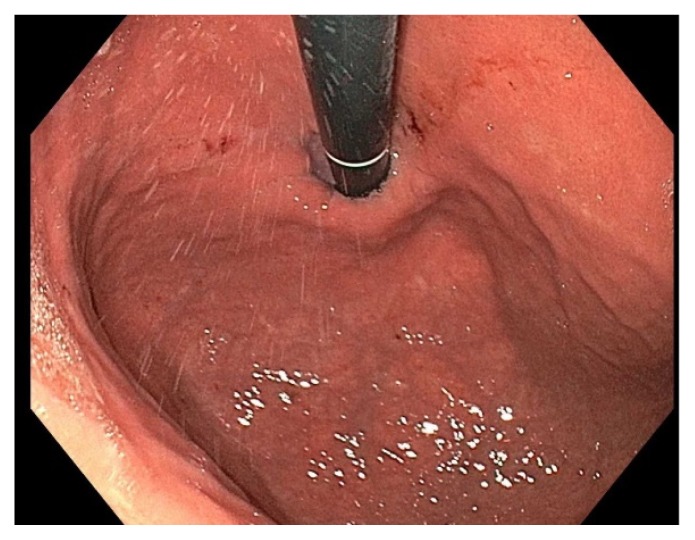
A retroflexed view of normal appearing stomach. Grossly no abnormalities were detected, hence random biopsies were obtained.

**Figure 2:**
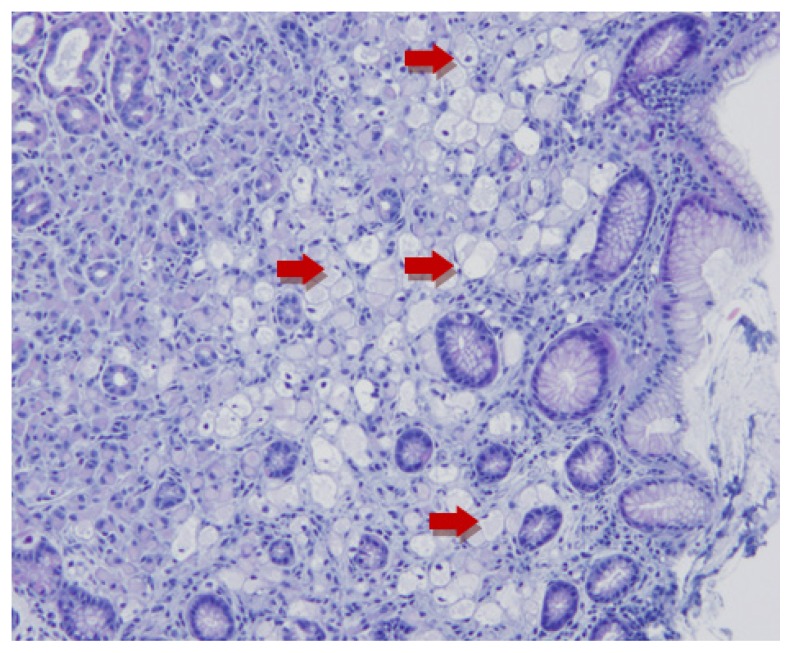
H&E stain on specimen obtained from biopsy during EGD exhibiting infiltrating adenocarcinoma poorly differentiated with mucinous and signet ring cell features (red arrows).

**Figure 3:**
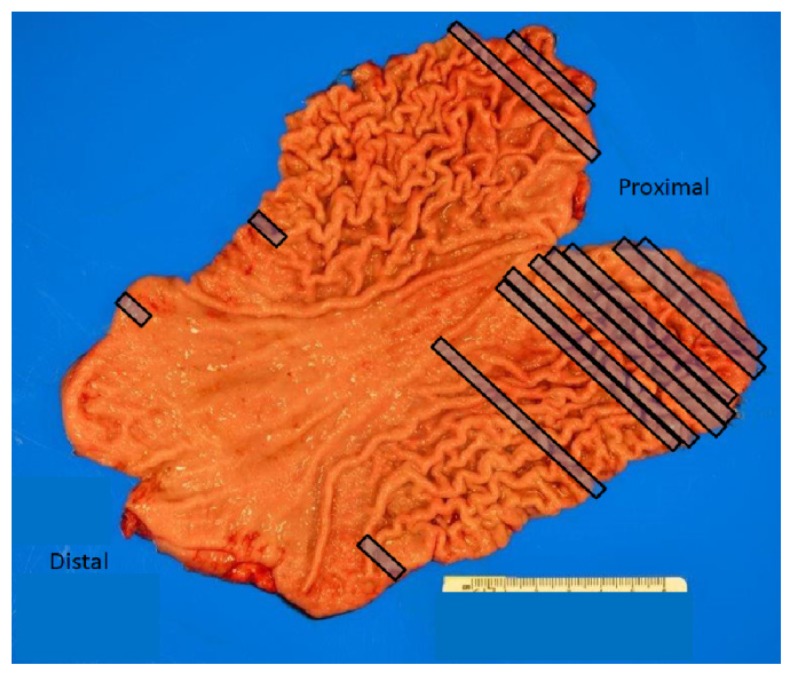
Gross specimen of the surgically resected stomach with rectangles highlighting the regions that had a positive foci of adenocarcinoma. (Note the longer rectangles indicated that more than 1 focus was discovered, however, they were difficult to pinpoint on the gross specimen.)

**Figure 4:**
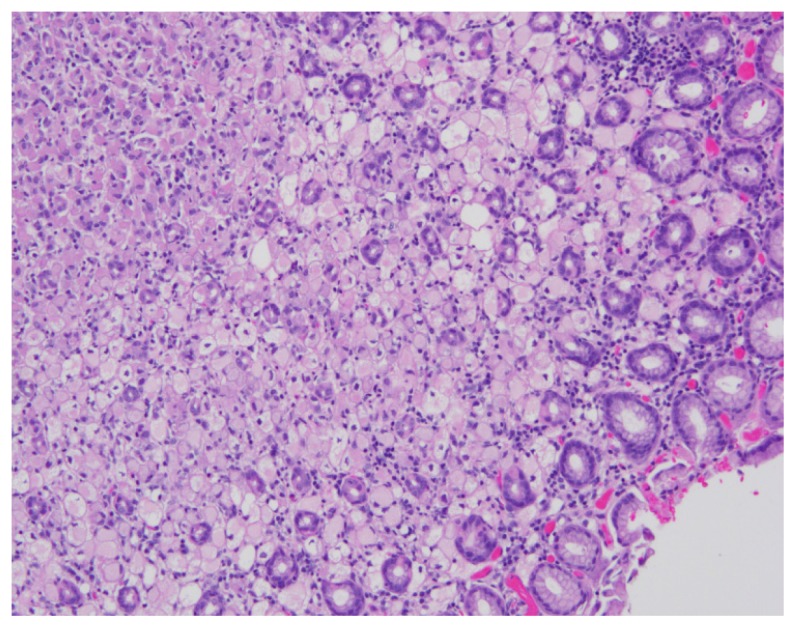
H&E stain of surgically resected specimen demonstrating adenocarcinoma.

**Figure 5:**
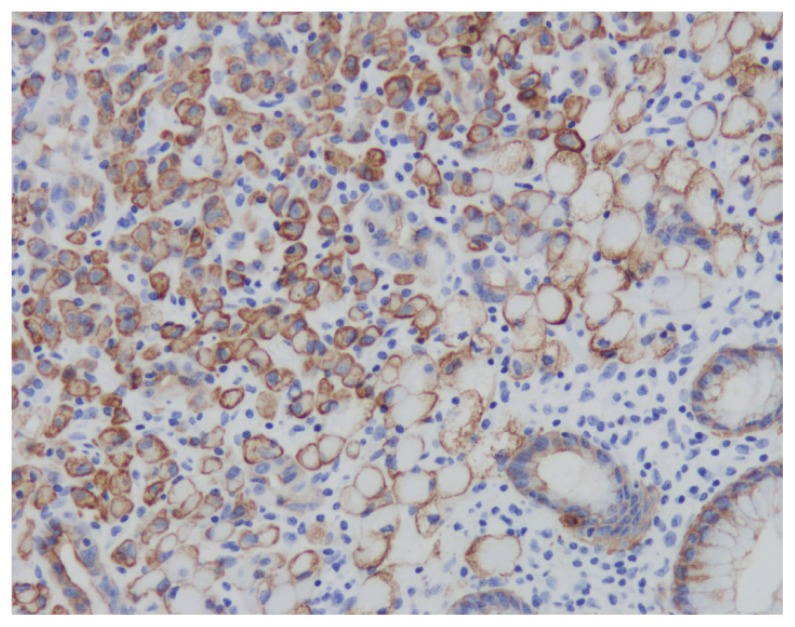
Pancytokeratin stain of surgically resected stomach showing adenocarcinoma.
